# Sensitivity of Yeast Mutants Deficient in Mitochondrial or Vacuolar ABC Transporters to Pathogenesis-Related Protein *Tc*PR-10 of *Theobroma cacao*

**DOI:** 10.3390/biology7020035

**Published:** 2018-06-13

**Authors:** Louise R. Barreto, Thayná Barreto, Sonia Melo, Cristina Pungartnik, Martin Brendel

**Affiliations:** Departamento de Ciências Biológicas, Laboratório de Biologia de Fungos, Centro de Biotecnologia e Genética, Universidade Estadual de Santa Cruz (UESC), Rodovia Jorge Amado, km 16, Ilhéus, Bahia, CEP 42665-000, Brazil; loubarreto88@gmail.com (L.R.B.); thaynabarreto1@gmail.com (T.B.); scmelo@uesc.br (S.M.); martinbrendel@yahoo.com.br (M.B.)

**Keywords:** *Saccharomyces cerevisiae*, ABC transporters, pathogenesis related protein, PR-10, oxidative stress

## Abstract

Pathogenesis-related proteins (PRs) are induced in plants after infection by pathogens and/or abiotic stress. Among these proteins, the family 10 (PR-10) influences the biosynthesis of secondary metabolites and shows antimicrobial ribonuclease activity. *Tc*PR-10p (Pathogenesis-related Protein 10 of *Theobroma cacao*) was isolated from resistant and susceptible *Moniliophthora perniciosa* cacao cultivars. Cell survival with *Saccharomyces cerevisiae* mutant lines deficient in ATP-binding cassette (ABC) transporter proteins indicated the influence on resistance to *Tc*PR-10p. Proteins of the ABC transport type are considered important in the process of resistance to antimicrobials and toxins. Thus, the objective of this work was to observe the sensitivity of ABC transporter yeast mutants in the presence of the *Tc*PR-10p. Chronic exposure of *S. cerevisiae* mitochondrial (BY*atm1Δ* and BY*mdl1Δ*) and vacuole (BY*nft1Δ*, BY*vmr1Δ*, BY*ybt1Δ*, BY*ycf1Δ* and BY*bpt1Δ*) ABC transporter mutants to *Tc*PR-10p (3 μg/mL, 0, 6, 12 and 24 h) was performed. Two *Tc*PR-10p sensitive strains (BY*mdl1Δ* and BY*nft1Δ*) were submitted to a fluorescence test with the fluorogenic dihydroethidium (DHE), to visualize the presence of oxidative stress in the cells. Oxidative stress-increased sensitivity was confirmed by flow cytometry indicating induced cell death either via apoptosis or necrosis. This yeast data combined with previous data of literature (of *M. perniciosa* sensitivity to *Tc*PR-10p) show that increased sensitivity to *Tc*PR-10p in these mutants could be due to the *Tc*PR10p-generated higher levels of intracellular reactive oxygen species (ROS), leading to increased cell death either via necrosis or apoptosis.

## 1. Introduction

Induced after infection by pathogenic agents and/or abiotic stress, pathogenesis related proteins (PRs) were first reported in tobacco mosaic virus infected tobacco plants [1,2,3]. Currently, it is known that PRs are induced in mono- and eu-dicotyledons when infected by fungi, viruses, bacteria, nematodes, or by insect attack, and in response to defence inducers, i.e., salicylic acid, jasmonic acid, and ethylene [4,5,6]. They are classified into 17 different families based on sequence homology, serological reactions, enzymatic reactions and biological activities [2,7].

Amongst the 17 different PR proteins, mechanisms of action and biological implications for members of the PR-10 family are not well comprehended, although they are proposed to have many roles in plants [8]. PR-10 proteins are expressed in conditions of pathogenesis or environmental stresses, suggesting that plant defences could be up-regulated under numerous biotic or abiotic stress disorders; specific proteins also demonstrate antimicrobial or ribonuclease activity [7]. Proteins classified as members of the PR-10 family are constitutively expressed in roots and other plant structures, i.e., stems, flower components, fruits, pollen grains and dried seeds, indicating a central role in growth and general plant development and differentiation [8,9,10,11].

PR-10 proteins (PR-10p) are encoded by multi-gene families, featuring the multifunctional aspect of these proteins, which have acquired different mutations and functions during evolution [12,13]. RNase activity of PR-10p was first described in *Panax ginseng* callus cell culture [14]. Their antifungal activity was identified in proteins expressed in pepper (*Capsicum annuum*), where hyphal extension of the pathogen *Hytophthora capsici* was inhibited, and in leaves of the lily *Lupinus albus*, where PR-10p was induced after infection by the fungus *Colletotrichum gloeosporioides* [15,16]. Recent studies identified DNase activity of PR-10p from rice [17] and grape [18], suggesting nuclease action in the programmed cell death process.

In order to analyse the processes that could involve PR-10 in plant-pathogen interaction, two cDNA libraries from cacao cultivars resistant and susceptible to *Moniliophthora perniciosa* (the fungus responsible for causing “witches’ broom” disease of cacao, *Theobroma cacao* L.) were constructed [19]. Thus, the gene related to the host resistance response of the cacao plant *Tc*PR-10 (Pathogenesis-Related Protein 10 of *Theobroma cacao*) could be isolated. *Tc*PR-10p is an acidic protein member of the PR-10 family. Similar genes were discovered in some gymnosperms and angiosperms and have been cloned and described in response to biotic and abiotic stress [20].

The first reported *Tc*PR-10p related activities were demonstrated by the presence of ribonuclease activity against the RNA of the fungus *Moniliophthora perniciosa* (Stahel), characterized as a dose- and time-dependent process. In vitro assays also showed that the presence of *Tc*PR-10p inhibited *M. perniciosa* growth, indicating antifungal activity [21]. Silva et al. identified in *M. perniciosa*, increased expression of proteins involved in processes of autophagy, resistance pathogens, and sterol production (important for maintenance of cellular homeostasis in fungi) when grown in contact with *Tc*PR-10p. These facts suggested that the *Tc*PR-10 gene could be used to increase the resistance of plants to pathogens [22]. Experimental results with mutants of the yeast *Saccharomyces cerevisiae* defect in DNA repair, membrane transporters, metal transporters, and antioxidant defense genes allowed a mechanism of action and active transport of *Tc*PR-10p to be proposed [19,21,22].

The yeast mitochondrial ABC transporter proteins (Mdl1p and Atm1p) are involved in the control of cellular iron homeostasis comprising the biogenesis of iron-sulfur clusters, as well as heme biosynthesis and peptide transport; while the vacuolar ABC transporter proteins (Nft1p, Ycf1p, Bpt1p, Vmr1p and Ybt1p), known as pumps that catalyze the transport of glutathione conjugates to the vacuole in the process of detoxifying the cell from a variety of compounds, including heavy metals, i.e., cadmium and arsenite, are involved in the process of transmembrane transport of substances, autophagy and vacuolar formation; all of them are ABC transporter proteins and important players in the process of resistance to stress, drugs and toxins [23,24,25].

Therefore, the objective of this work was not only to continue research on the membrane transport of *Tc*PR-10p, but also to contribute to the improvement in understanding of the effects of *Tc*PR-10p inside the fungal cells. For this purpose, due to the difficulty in manipulating the filamentous fungus *M. perniciosa*, we used mutants of the single cell eukaryote *S. cerevisiae* lacking genes encoding mitochondrial (BY*mdl1*Δ and BY*atm1*Δ) and vacuolar (BY*nft1*Δ, BY*ycf1*Δ, BY*bpt1*Δ, BY*vmr1*Δ and BY*ybt1*Δ) ABC transporter proteins.

## 2. Materials and Methods

### 2.1. Obtaining the TcPR-10 Protein

The cocoa gene *Tc*PR-10, isolated from the cDNA library of interaction *T. cacao* × *M. perniciosa*, (accession number ES439858), was cloned into expression vector pET28a and the recombinant protein *Tc*PR-10p expressed in *Escherichia coli* BL21 (DE) as described by Gesteira et al. [19]. *Tc*PR-10p was purified and quantified using Bradford assay with a minimum of three different replicates prior to realizing the experiments.

### 2.2. Growth Conditions of Yeast S. cerevisiae

The *S. cerevisiae* strains used in this work are listed in Table 1. Media, solutions and buffers were prepared according to Burke et al. [26]. To obtain yeast cells in stationary growth phase (STAT cells, density of approximately 2 × 10^8^ cells/mL), stock aliquots in glycerol were inoculated in rich medium (YPD—10 g/L extract yeast; 20 g/L Bacto Peptone; 20 g/L dextrose) and incubated under constant aeration in a gyratory shaker (New Brunswick, G-76) for 2–3 days, or in solid medium YPD (YEL + 20 g/L agar) at 28 °C. Cells in exponential growth phase (LOG cells) were obtained by 5 to 12 h of incubation at 28 °C under constant aeration in a gyratory shaker (New Brunswick, G-76, 180 rpm), reaching 1 to 2 × 10^7^ cells/m. A culture was considered in LOG phase when at least 30% of the cells were budding. To ascertain their respiratory competence and eliminate spontaneously accumulated petites, all strains were pre-grown on solid YPG medium (YPD in which glucose was replaced by 2% glycerol) before being used for experimentation. Experiments were done at least three times in triplicate.

### 2.3. Yeast Survival after TcPR-10p Exposure

For treatment with *Tc*PR-10p, cells in LOG cells (OD_660_ between 1.2 to 2.4 × 10^7^ cells/mL) were centrifuged at 5000 g for 5 min and suspended in 50 mM sodium phosphate buffer (pH 7.4). Cell titre of approximately 10^5^ cells/mL was determined by absorbance reading (660 nm) in a Spectrophotometer V-1600^®^ (Roctec Technology, Hong Kong, China) [26]. Aliquots were treated with the same dose as described in previous studies, i.e., 3 μg/mL *Tc*PR-10p at 25 °C for exposure times 0, 1.5 3, 6, 12 and 24 h. The samples were properly diluted, plated on YPD agar and tested for cell survival after 3 days of incubation at 28 °C.

Cell survival was calculated once counting the number of colonies grown on the plates following 3 d of growth at 28 °C and calculating the fraction of *Tc*RP-10p-exposed survival in relation to the zero dose and expressed as a percentage (N/No × 100). The measurement of the effect of *Tc*PR-10p using the percentage survival vs. dose with a logarithmic ordinate was visualized using a typical inactivation kinetics curve, where the rate of decrease of the number of active single yeast cells with respect to dose is proportional to the number of active cells remaining at that dose level [27]. When killing follows single hit kinetics, the inactivation curve is exponential, and the graph will yield a linear curve with a negative slope in a semi-log plot. Survival is presented in a semi-log graph to enable rapid estimation of the dose reduction factor [28]. Statistical analysis between parallel experiments was performed using the standard deviation, using the GraphPad Prism^®^ program (GraphPad Software Incorporation, San Diego, CA, USA).

Graphs were generated using the GraphPad Prism^®^ program (GraphPad Software Incorporation, San Diego, CA, USA); error bars represent the standard deviations of at least three independent experiments, in triplicate.

### 2.4. Fluorescence Assay of TcPR-10p Yeast Mutants

Mutant strains in LOG phase were treated with 3 μg/mL of *Tc*PR-10p for 6 and 24 h. Thereafter, cells were washed with saline solution (NaCl 0.9%) and suspended in a final volume of 1 mL. A stock solution (1 mg/mL) of the fluorogenic probe dihydroethidium (DHE, SIGMA-ALDRICH^®^, St. Louis, MO, USA) was prepared by dissolving it in dimethyl sulfoxide (Sigma, St. Louis, MO, USA). One mL of yeast cells was stained with the addition of 1 μL of stock solution, mixed by inversion, incubated for 30 min at 28 °C, washed three times with saline, and re-suspended in 100 μL of saline. An aliquot was used to determine the oxidative/reductive stress of the cells. Cytosolic DHE can be oxidized by reactive oxygen species (ROS, i.e., singlet oxygen, hydroxyl radicals, superoxide, hydroperoxides and peroxides) to form ethidium, which intercalates with the cellular DNA and brightly fluoresces red (λ = 605 nm) [29]. ROS induction was observed by fluorescence microscopy DMRA2 (Leica^®^, Wetzlar, Germany) using a DHE filter. Images were captured using a 40× objective under bright field and using fluorescent filters with the IM50 software (Leica^®^). Photos represent one sample from at least three independent experiments.

### 2.5. Flow Cytometry

Yeasts mutants that showed sensitivity at the survival curve and presence of oxidative stress by the DHE assay underwent cell death assessment through Cell Cytometry using two different dyes to Alexa Fluor^®^ 488 annexin V, which allows the labelling of phosphatidylserine (PS), exposed by the cell during the apoptosis process, and propidium iodide (PI), an impermeable dye that allows the integrity of the cytoplasmic membrane to be evaluated. LOG phase cells (OD660 between 1.2 to 2.4 × 10^7^ cells/mL) were treated with *Tc*PR-10 for 24 H (3 μg/mL), then cells were washed with 50 mM sodium phosphate buffer (pH 7.4), centrifuged at 5000 g for 5 min. This process was repeated 3 to 4 times, and cells were suspended in the final volume of 500 μL with 50 mM sodium phosphate buffer (pH 7.4). The cells were then labelled, according to the manufacturer’s instructions, using the Anexinn V-FITC/PI kit (Beckman Coulter) and read (50,000 acquisitions/tube) on the FC 500 flow cytometer (Beckman Coulter) in order to evaluate the percentage of cells in apoptosis. Experiments were done in triplicate.

### 2.6. Analysis of Results

All experiments were performed in triplicate and expressed with mean and standard deviation. Mean-variance analysis tests were performed by the Graph Pad Prism^®^ program (Graph Pad Software, Inc., San Diego, CA, USA).

## 3. Results

### 3.1. Yeast Survival after Exposure to TcPR-10p

**Figure 1:**
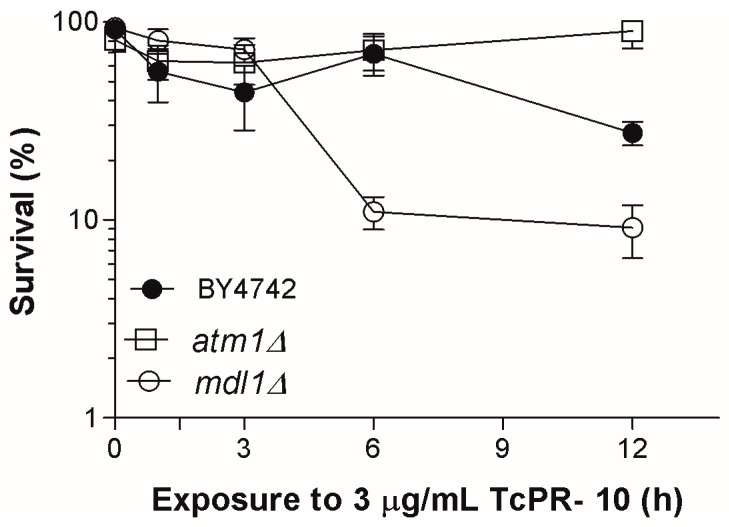
Survival of LOG phase mitochondrial ABC transporter-deficient *S. cerevisiae* mutants when exposed to 3 μg/mL of *Tc*PR-10p. Result expressed with mean deviation and standard deviation of three independent experiments.

The mitochondrial transporter ABC proteins Mdl1p and Atm1p are implicated in the regulation of cellular iron homeostasis, comprising the biogenesis of iron-sulphur clusters [24]. LOG phase cells of the BY*mdl1*Δ mutant exposed to 3 μg/mL *Tc*PR-10p exhibit significant increases in sensitivity, while equally treated BY*atm1*Δ mutant cells were more resistant when compared to the equally treated wild type (WT) (Figure 1). 

**Figure 2:**
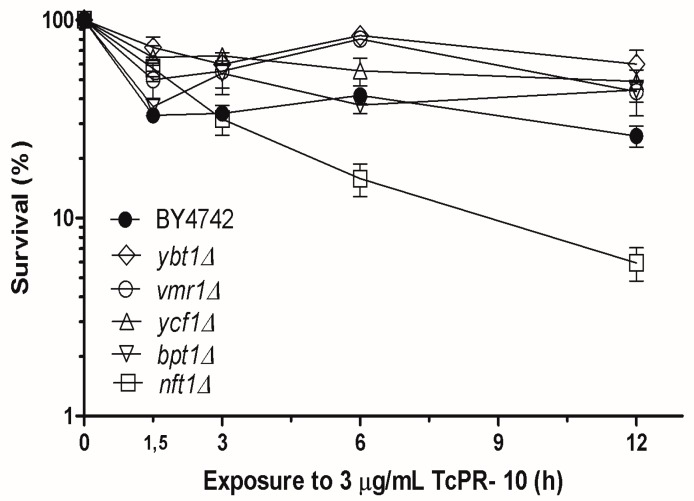
Survival of LOG phase wild-type (WT) and five isogenic yeast *Saccharomyces cerevisiae* vacuolar ABC transporter- deficient mutants exposed to 3 μg/mL of *Tc*PR-10p (0 to 12 h). Results expressed with mean deviation and standard deviation of three independent experiments.

Survival graph of the five *Tc*PR-10p -exposed mutant strains BY*nft1*Δ, BY*ycf1*Δ, BY*bpt1*Δ, BY*vmr1*Δ and BY*ybt1*Δ, each lacking a specific ABC transporter protein located in the vacuolar membrane is shown in Figure 2. The survival profile of LOG phase yeast cells exposed to 3 μg/mL *Tc*PR-10p in show that only one of the five tested mutant, BY*nft1*Δ presents significantly increased sensitivity when compared to equally treated WT strain.

**Figure 3:**
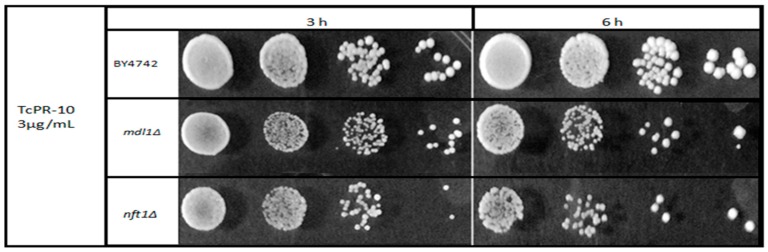
Sensitivity of yeast mutants lacking either mitochondrial (Mdl1p) or vacuolar (Nft1p) ABC transporter proteins after 3 and 6 h exposure to 3 μg/mL *Tc*PR-10p.

To facilitate the perception of sensitivity, a drop survival curve was performed with the two mutant strains tested that showed higher sensitivity after exposure with 3 μg/mL of *Tc*PR-10p when compared to the WT BY4742 strain. The test clearly shows greater sensitivity of the two mutant strains after 6 h of exposure (Figure 3).

### 3.2. Yeast Sensitive Mutants Show Increased ROS Levels Induced by TcPR-10p

**Figure 4:**
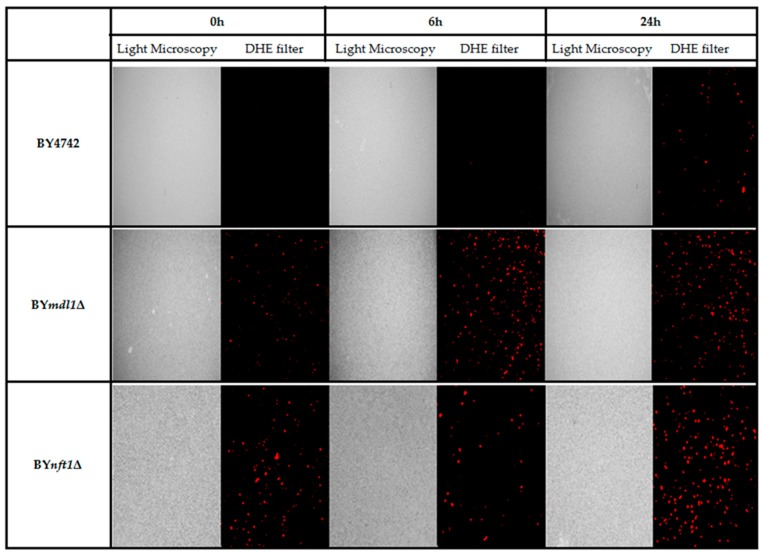
*Tc*PR-10p induced ROS in isogenic *S. cerevisiae* strains: WT, BY*mdl1Δ* and BY*nft1Δ* treated with 3 μg/mL *Tc*PR-10p for 6 and 24 h and observed by fluorescence microscopy (DHE filter).

Specific sensitivity of two out of seven ABC transporter mutants could be due to the fact that *Tc*PR-10p is able to induce oxidative stress [20,21,22]. The images obtained after marking with DHE show the increased ROS generated after exposure to *Tc*PR-10p for 6 and 24 h of yeast mutants BY*mdl1*Δ and BY*nft1*Δ strains when compared to WT (Figure 4).

The stress responses of WT BY4742 and mutant BY*mdl1*Δ increases with exposure time as can be observed by the correlation of free radical presence with time of exposure. In contrast, the strain BY*nft1*Δ (disabled in the process of autophagy) already has high initial ROS levels but still shows even higher ROS levels after 24 h of *Tc*PR-10p exposure.

### 3.3. Flow Cytometry Assay of TcPR-10p Sensitive Yeast Mutants

**Figure 5:**
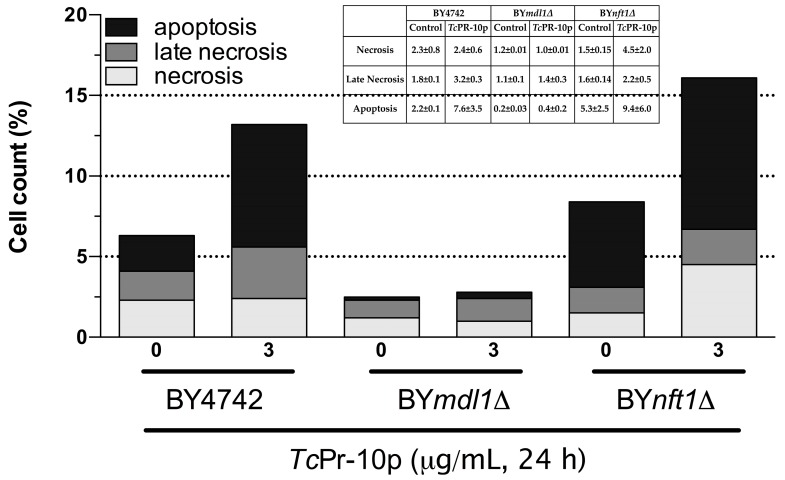
Flow cytometry assay of isogenic *S. cerevisiae* yeast strains treated with 3 μg/mL *Tc*PR-10p for 24 h. WT BY4742; BY*mdl1*Δ; and BY*nft1*Δ. (one figure representative of scatter plots formed from bi-parametric analysis).

The increased sensitivity associated with oxidative stress induced by *Tc*PR-10p in ABC transporter mutants with different phenotypes and different ABC transporter membrane location BY*mdl1*Δ (mitochondria) and BY*nft1*Δ (vacuole) could trigger different metabolic responses. Therefore, we analyzed via the flow cytometry technique, DNA fragmentation (apoptosis—Anexin V), cell viability (necrosis—PI), and double labelling with annexin V conjugated to FITC and propidium iodide (apoptosis and necrosis) (Figure 5, Supplementary Table S1). In the mutant yeast strains BY4742 and BY*md1l*Δ, an increased number of apoptotic cells after *Tc*PR-10p was observed when compared to the respective controls. For the BY*nft1*Δ mutant it was possible to observe the presence of three times more cells in necrosis when exposed to *Tc*PR-10p than the control group, according to Figure 5 and Supplementary Table S1.

## 4. Discussion

The pathogenesis-related protein PR-10 is produced in plants under conditions of stress or after infection. *Tc*PR-10p is a member of the acidic protein family PR-10 [20]. Isolated from *M. perniciosa* resistant *T. cacao* cultivars it was characterized as having RNase activity, causing oxidative stress and promoting the death of pathogens in a response of plant defence [19,30,31,32].

The cellular plasma membrane and membranes of different organelles contain transporter proteins of the ABC type that are involved in the processes of resistance and sensitivity of the pathogen to certain host defence toxins [33]. Yeast mutants lacking ABC transporters of the plasma membrane were tested for sensitivity to *Tc*PR-10p in an attempt to elucidate the transport routes and mode of action of this protein inside the cells [21,22]. Among the tested ABC transporter-deficient mutant strains, BY*snq2*Δ and BY*pdr11*Δ exhibited a *Tc*PR-10p sensitivity profile as compared to the isogenic WT. These results indicate that the sensitivity of the fungus to *Tc*PR-10p comprises numerous biochemical routes, elucidating the probable types of action of this antifungal protein [21,22].

As for our current research, we used WT strain BY4742 and different isogenic mutants, each lacking one ABC transporter-coding gene of different subfamilies located in mitochondria (BY*atm1*Δ and BY*mdl1*Δ) or vacuole (BY*nft1*Δ, BY*ycf1*Δ, BY*bpt1*Δ, BY*vmr1*Δ and BY*ybt1*Δ) with the objective of evaluating the possible interaction of intracellular *Tc*PR-10p, imported via a plasma membrane with the WT composition of ABC transporters, with mitochondria and vacuoles lacking one defined ABC transporter protein. Such organelle membrane deficiency could lead to an altered phenotype, revealed as a change in yeast cell sensitivity to *Tc*PR-10p [34].

Both Atm1 and Mdl1 proteins are located in the inner mitochondrial membrane with the ATP binding domain oriented to the matrix, and have an important role in mitochondrial export [24,35]. Atm1p is a cellular iron homeostasis regulator, and its absence causes an increased accumulation of iron and oxidized glutathione in a stress process caused by a Fenton reaction [23,36]. The presence of Mdl1p is able to complement the absence of Atm1p [37]. This feature keeps the mitochondrial export in mutant BY*atm1*Δ active, which may explain the observed WT-like or more resistant survival profile of *Tc*PR-10p treated *atm1*Δ mutant (Figure 1).

Increased sensitivity of the BY*mdl1*Δ mutant to *Tc*PR-10p may be related to characteristics of the absence of this protein related to oxidative stress. Mdl1p accounts for the export of peptides produced after proteolysis of mitochondrial proteins; and also plays a significant role in the regulation of cellular resistance to oxidative stress [38]. BY*mdl1*Δ mutant shows a 40% reduction in the release of peptides generated by mitochondrial proteases [24]. Besides having binding sites for fatty acids, flavonoids, cytokines and sterols, *Tc*PR-10p has been associated with ribonuclease activity. An increase in degraded RNA promotes intracellular oxidative stress by the formation of free radicals that may also act as marker for cell death [39]. Therefore, the combination of increased malformed peptides (accumulated inside the organelle leading to stress signals due to the absence of peptide export) and the presence of free radicals (formed by accumulation of degraded RNA) may both be responsible for the increased cell death after 6 h of *Tc*PR-10p exposure in the Mdl1p-lacking mutant (Figure 1).

Vacuoles are compartments participating in important physiological processes of yeast cells, such as: storage of nutrients, autophagy, degradation of cell compounds, and also recycling of proteins [25,40]. The ABC transporter-dependent vacuolar sequestration of unwanted cellular molecules is an important recycling and detoxification mechanism [40]. Applying phylogenetic analysis Wawrzycka et al. [41] divided vacuole-located ABC transporter type proteins into three groups: Cluster 1: Ybt1p (bile acid transporter and negative regulator in vacuole formation) and its homologue Vmr1p (transport of conjugated DNP-S-glutathione, export S-(2,4-dinitrophenyl)-glutathione); Cluster 2: Ycf1p and its homologue Bpt1p (pumps responsible for catalysing glutathione transport and detoxifying the cell of a variety of heavy metals, i.e., cadmium and arsenate), and Nft1p (responsible for autophagy); and Cluster 3: comprises only Yor1p (not included in our study). The same study showed the existence of homology between the Vmr1 and Ybt1 proteins and their functional interactions [42,43]. 

Out of five vacuolar yeast mutants, four had slightly increased resistance to *Tc*PR-10p under the tested conditions when compared to the respective WT (Figure 2). Yeast Ybt1p is a negative regulator of vacuole formation, and in its absence, a larger number of vacuoles are formed, reducing stress [41]. Ybt1p and Vmr1p are homologues; the same study showed the existence of homology between the Vmr1 and Ybt1 proteins and their functional interactions, and a conjugate of a protein was used as substrate to mediate the transport of another [42,43]. Likewise, Ycf1p reduces basal level of oxidative stress reaction in *S. cerevisiae* [43]. This WT-like resistance of each one of the vacuole ABC transporter-lacking mutants BY*ycf1*Δ, BY*bpt1*Δ, BY*vmr1*Δ and BY*ybt1*Δ that are homologous to each other was expected (Figure 2).

In contrast, the absence of vacuolar Nft1p leads to a decrease in autophagic activity, which is important for cell degradation processes, and maintenance of homeostasis and lipid transport to the expanding membrane during the process of membrane closure [25,40]. The ribonuclease activity of *Tc*PR-10p degrades nucleic acids, thereby inducing oxidative stress [21,22]. Thus, with reduced autophagy, the recycling of intracellular accumulated unwanted stress-causing compounds is decreased; this sensitizes only BY*nft1*Δ mutant cells to the ROS generated by *Tc*PR-10p, leading to the observed increased cell death (Figure 2 and Figure 3).

In order to confirm that ROS generated by *Tc*PR-10p could be the culprit and influence sensitivity of both BY*mdl1*Δ and BY*nft1*Δ mutants, DHE was used to observe induced ROS formation after *Tc*PR-10p exposure under same conditions of the survival (Figure 4). The proportional increase of ROS in BY*mdl1*Δ mutant cells to *Tc*PR-10p exposure time is clearly observable under fluorescence microscopy images (Figure 4). In BY*nft1*Δ mutant cells, with reduced ability of autophagy, it is possible to observe increased oxidative stress at all exposure times (6 and 24 h). Yet, the reduction of autophagic activity [44] seems to be related to the presence of high levels of ROS in the BY*nft1*Δ mutant (Figure 4), and to sensitivity observed in the survival tests (Figure 2 and Figure 3). 

Members of the PR-10 family of proteins have been reported for playing a crucial role during the attack of a pathogen by activating apoptotic processes in the pathogen, thus limiting the success of the invasion [45]. Indeed, in yeast strains BY4742 and BY*md1l*Δ, an increased number of apoptotic cells after *Tc*PR-10p is observed when compared to the respective controls, while for the BY*nft1*Δ mutant, it is possible to observe the presence of three times more cells in necrosis when exposed to *Tc*PR-10p than the control group according to Figure 5. 

Until now, there is only a limited number of factors, i.e., ageing, mating, or exposure to killer toxins and the application of acetic acid, antifungal peptides, hydrogen peroxide, and oxygen radicals, known to induce programmed cell death in yeast [46,47]. Our data obtained from this study suggest that absence of Mdl1p (mitochondrial ABC transporter protein responsible for monitoring resistance to oxidative stress) leads to cellular death by apoptotic pathway as presence of *Tc*PR-10p causes increased oxidative stress verified by high levels of intracellular ROS (Figure 4 and Figure 5), while in BY*nft1*Δ mutant leads to cellular death by necrosis. Numerous studies on apoptosis point to mitochondria as the main mediator of cell death. This organelle integrates cell death stimuli, inducing mitochondrial permeabilization, which results in release of pro-apoptotic molecules [48]. In addition to leaking molecules, induced collapse of potential of the inner mitochondrial membrane leads to loss of cellular homeostasis, interrupting ATP synthesis and increasing ROS production [49,50]. 

## 5. Conclusions

Apart from well-confirmed RNase activity of PR-10p in different organisms, including *M. perniciosa*, studies that identified DNase activity in rice and grape likewise suggested nuclease action in apoptosis. Additionally, many proteins associated with detoxification, autophagy, or that were involved in mechanisms for maintaining fungal homeostasis (such as ergosterol biosynthesis) were induced by *Tc*PR-10, as shown by proteomics analysis, a fact that is coherent with the antifungal activity of *Tc*PR-10. Recently, we could also observe changes in cellular infrastructure after induced endoplasmic reticulum (ER) stress in *M. perniciosa*, i.e., remodelling of the ER under stress, formation of ER-phagy, and induction of apoptosis after severe ER stress. 

Mutants of *S. cerevisiae* that lack mitochondrial ABC transporter protein Mdl1p or vacuolar ABC transporter Nft1p showed increased stress sensitivity to *Tc*PR-10p exposure when compared to the isogenic WT. However, this was most probably due to different biochemical mechanism of action, since *Tc*PR-10p treated BY*md1l*Δ mutant showed increased numbers of apoptotic cells, while for the equally treated BY*nft1*Δ mutant a higher number of necrotic cells were observed. This yeast data, combined with previous data of literature (of *M. perniciosa* sensitivity to *Tc*PR-10p), shows that yeast increased sensitivity to *Tc*PR-10p could be due to the higher levels of intracellular ROS generated by the *Tc*PR10p, in these mutants, which would lead to increased cell death either via necrosis or apoptosis.

This study attempts to clarify the mode of action of the *T. cacao* defence protein *Tc*PR-10p using the yeast *S. cerevisiae* as a research model. The obtained data may help us to understand the effects of *Tc*PR-10p on multicellular fungal phytopathogens such as *M. perniciosa*, a basidiomycete fungus that, by causing “witches’ broom” disease in *T. cacao*, leads to substantial economic loss in cacao production. Nevertheless, supplementary experiments in planta are necessary to understand the mechanisms of resistance of this fungus to the plant defence mechanism. A better understanding of the fungal resistance to oxidative stress may give us the means to weaken the efficiency of this process and enhance the plant’s chances to fight fungal infection.

## Figures and Tables

**Table 1:** Yeast strains, genotype and related function.

Strains *	Genotype	Name Description/Cellular Component **
BY4742 (WT)	MAT*α his3*∆1 *leu2*∆0 *lys2*∆0 *ura3*∆0	Wild type for ABC transporters
BY*mdl1*Δ	Same as WT, YLR188w deleted	Multi-Drug resistance-Like/Mitochondrial inner membrane half-type ABC transporter; mediates export of peptides generated upon proteolysis of mitochondrial proteins; plays a role in the regulation of cellular resistance to oxidative stress
BY*atm1*Δ	Same as WT, YMR301c deleted	ABC Transporter, Mitochondria/Mitochondrial inner membrane ATP-binding cassette (ABC) transporter; exports mitochondrially synthesized precursors of iron-sulfur (Fe/S) clusters to the cytosol
BY*ybt1*Δ	Same as WT, YLL048c deleted	Yeast Bile Transporter/Transporter of the ATP-binding cassette (ABC) family; negative regulator of vacuole fusion
BY*nft1*Δ	Same as WT, YKR103w deleted	New Full-length MRP-type Transporter/Transporter of the ATP-binding cassette (ABC) vacuole; multidrug resistance-associated protein
BY*yct1*Δ	Same as WT, YLL055w deleted	Yeast Cysteine Transporter/Transporter of the ATP-binding cassette (ABC) vacuole; High-affinity cysteine-specific transporter
BY*bpt1*Δ	Same as WT, YLL015w deleted	Bile Pigment Transporter/ABC type transmembrane transporter of MRP/CFTR family; found in vacuolar membrane, involved in the transport of unconjugated bilirubin and in heavy metal detoxification via glutathione conjugates, along with Ycf1p
BY*vmr1*Δ	Same as WT, YHL035c deleted	Vacuolar Multidrug Resistance/Vacuolar membrane protein; involved in multiple drug resistance and metal sensitivity; ATP-binding cassette (ABC) family member involved in drug transport

* All strains are from EUROSCARF; ** http://www.yeastgenome.org.

## Supplementary Materials

The following are available online at http://www.mdpi.com/2079-7737/7/2/35/s1: Table S1: Statistical analysis of data of Figure 5. Two way ANOVA, Sidak’s and Tukey’s multiple comparison test.

## Abbreviations

WT, wild type like strain. *Tc*PR-10p, pathogenesis-related protein 10 of *Theobroma cacao*. PR, pathogenisis-related protein. ABC transporter, ATP binding cassette transporter. MRP/CFTR, cystic fibrosis transmembrane conductance regulator, ATP-binding cassette, sub-family C, CFTR is a member of the MRP (multidrug resitance proteins) subfamily that is involved in multi-drug resistance. PI, propidium iodide. FITC, Fluorescein isothiocyanate.
