# Supplementary material for: Sensitivity of Yeast Mutants Deficient in Mitochondrial or Vacuolar ABC Transporters to Pathogenesis-Related Protein TcPR-10 of Theobroma cacao
Source: Biology (Basel). 2018 Jun 13;7(2):35. doi: 10.3390/biology7020035 (PMC6022951; doi:10.3390/biology7020035)
Supplement: Supplementary file 1 [file biology-07-00035-s001.pdf]

Supplementary Table S1.

Statistical analysis of data of Figure 5. Two way ANOVA, Sidak's and Tukey's multiple comparison test.

	**necrosis x late necrosis**	**necrosis x apoptosis**	**late necrosis x apoptosis**
**BY4742**	ns	ns	ns
**BY4742 Tc**	ns	*	ns
**BY*mdl1***	ns	ns	ns
**BY*mdl1* Tc**	ns	ns	ns
**BY*nft1***	ns	ns	ns
**BY*nft1* Tc**	ns	*	**

	**BY4742 x BY4742 Tc**	**BY*mdl1* x BY*mdl1* Tc**	**BY*nft1* x BY*nft1* Tc**
**necrosis**	ns	ns	ns
**late necrosis**	ns	ns	ns
**apoptosis**	*	ns	ns

	**BY4742**	**BY4742 Tc**	**BY*mdl1***	**BY*mdl1* Tc**	**BY*nft1***	**BY*nft1* Tc**
**BY4742**	ns	ns	ns	ns	ns	late necrosis x apoptosis, *
**BY4742 Tc**	ns	ns	apoptosis x apoptosis, *	ns	ns	ns
**BY*mdl1***	ns	ns	ns	ns	ns	necrosis x apoptosis, *<br>late necrosis x apoptosis, *<br>apoptosis x apoptosis, **
**BY*mdl1* Tc**	ns	ns	ns	ns	ns	necrosis x apoptosis, *<br>late necrosis x apoptosis,*<br>apoptosis x apoptosis, *
**BY*nft1***	ns	ns	ns	ns	ns	necrosis x apoptosis, *<br>late necrosis x apoptosis, *

**BY*nft1* Tc**	ns	ns	ns	ns	ns	ns
